# Diagnosis and management of a patient with primary pulmonary diffuse large B-cell lymphoma: A case report and review of the literature

**DOI:** 10.3892/etm.2014.1797

**Published:** 2014-06-20

**Authors:** AI-GUI JIANG, XIAO-YAN GAO, HUI-YU LU

**Affiliations:** Department of Respiratory Medicine, Taizhou People’s Hospital, Taizhou, Jiangsu 225300, P.R. China

**Keywords:** large B-cell lymphoma, primary, pulmonary, transbronchial needle aspiration

## Abstract

Primary pulmonary lymphoma (PPL) is an uncommon type of non-Hodgkin’s lymphoma. The majority of PPLs are of low-grade, mucosa-associated lymphoid tissue type. Primary pulmonary diffuse large B-cell lymphoma (DLBCL) is extremely rare, and prompt diagnosis may be challenging since its clinical symptoms and signs are nonspecific. Although the clinical features, diagnostic procedures, optimal management and prognostic factors of this disease have not yet been well defined, open thoracotomy and chest computed tomography (CT)-guided percutaneous biopsy are the preferred methods used in previous studies. In the present case report, the diagnosis and management of a patient with primary pulmonary DLBCL is reported. A 68-year-old patient was admitted to hospital in May 2013, with complaints of shortness of breath and intermittent wheezing and a cough associated with the production of small amounts of phlegm. Following admission, chest CT scans revealed a mass in the right middle lobe with ground-glass opacities at the lesion margins, as well as air bronchograms in the areas of consolidation. Bronchoscopy was performed and revealed an endobronchial lesion and partial stenosis in the distal end of the middle segment bronchus. Transbronchial needle aspiration (TBNA) of the right hilar lymph node, as well as endobronchial biopsy, was performed. The patient was diagnosed with primary pulmonary DLBCL by subsequent histopathological and immunohistochemical analysis of biopsy specimens collected via TBNA. Following the final diagnosis, standard treatment with CHOP chemotherapy resulted in significant clinical and radiological response and the patient remained in remission 8 months later. These results indicate that TBNA may be an effective method for the diagnosis of primary pulmonary DLBCL.

## Introduction

Primary pulmonary lymphoma (PPL) is an uncommon type of non-Hodgkin’s lymphoma (NHL). The vast majority of PPLs are of low-grade, mucosa-associated lymphoid tissue (MALT) type. Primary pulmonary diffuse large B-cell lymphoma (DLBCL) is particularly rare and occurs only in 10% cases of primary pulmonary NHL (1). The rapid diagnosis of primary pulmonary DLBCL is challenging since the clinical symptoms and signs are nonspecific. Although the clinical features, diagnostic procedure, optimal management and prognostic factors of this disease have has yet to be clearly elucidated, previous studies have indicated that open thoracotomy or chest computed tomography (CT)-guided percutaneous biopsy are the preferred methods of diagnosis (2). To the best of our knowledge, the positive rate of diagnosis via direct bronchoscopic biopsy is low, and has been reported in only ~10% of patients in previous studies (3–5). In the present case study, the diagnosis of a patient with primary pulmonary DLBCL via transbronchial needle aspiration (TBNA) is reported.

## Case report

This study was performed in accordance with the Declaration of Helsinki and approved by the Ethics Committee of Taizhou People’s Hospital, (Taizhou, Jiangsu, China). Written informed consent was obtained from the patient. A 68-year-old male patient was admitted to the Department of Respiratory Medicine, Taizhou People’s Hospital in May 2013, with complaints of shortness of breath and intermittent wheezing and a cough associated with the production of small amounts of phlegm that had existed for one and a half years. The patient was a nonsmoker with no prior history of lung disease and no exposure to occupational or dust hazards. Following admission, physical examination revealed a body weight of 62 kg, height of 172 cm, body temperature of 36°C, pulse of 92 bpm, respiratory rate of 21 bpm and blood pressure of 130/80 mmHg. No focal findings were observed on examination, particularly of the skin. In addition, no palpable lymph nodes and no hepatosplenomegaly was observed. The patient was slightly haggard in appearance, with no cyanosis of the lips. Bilateral respiratory movements were identical, vocal fremitus was equal bilaterally and dry rales were audible in the right lung.

**Figure 1:**
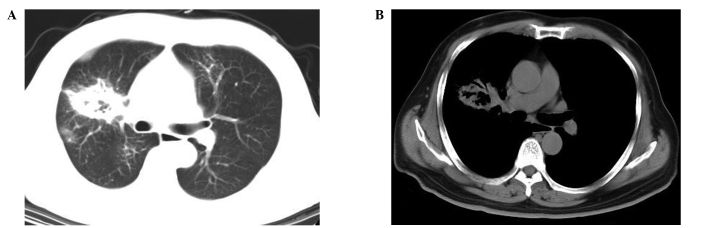
Computed tomography scan of the (A) lung window and (B) mediastinal window, revealing a mass in the right middle lobe with ground-glass opacities around it and air-filled bronchi in the consolidation.

**Figure 2:**
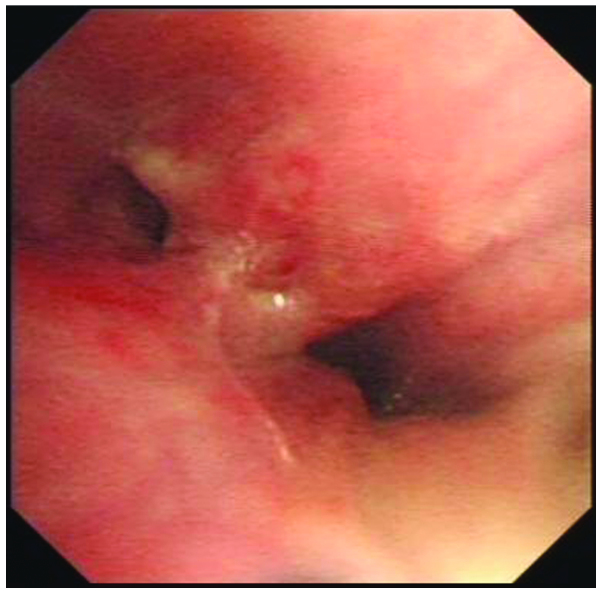
Bronchoscopy revealed an endobronchial lesion and partial stenosis in the distal end of the middle segment bronchus.

**Figure 3:**
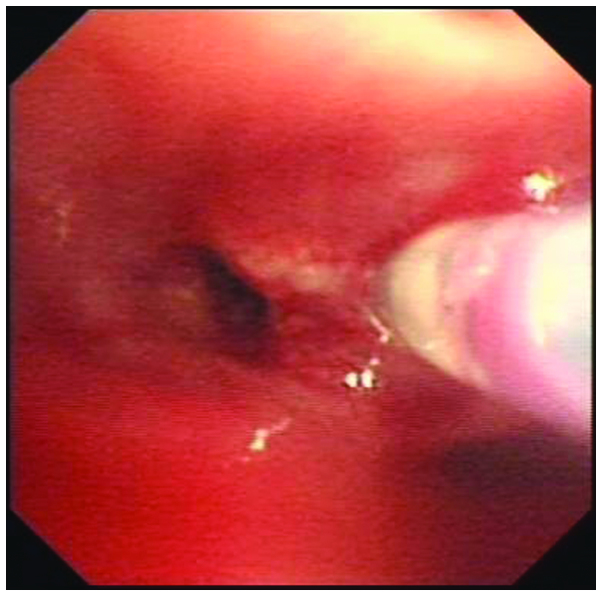
Transbronchial needle aspiration of the right hilar lymph node biopsy.

**Figure 4:**
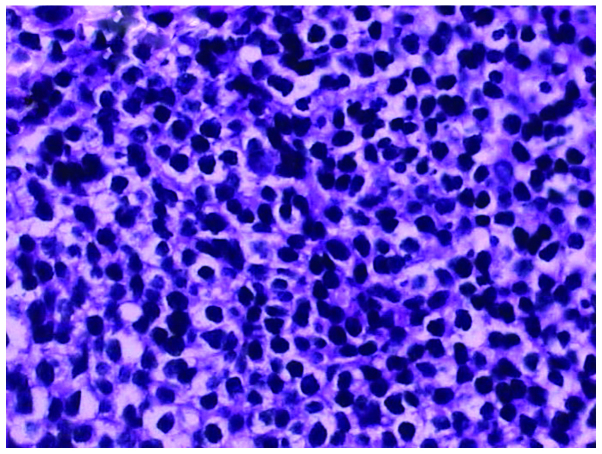
Histopathological analysis revealed an aggressive large cell lymphoma, with scattered large, oddly shaped nuclei resembling Reed-Sternberg cells (hematoxylin and eosin; magnification, ×200).

**Figure 5:**
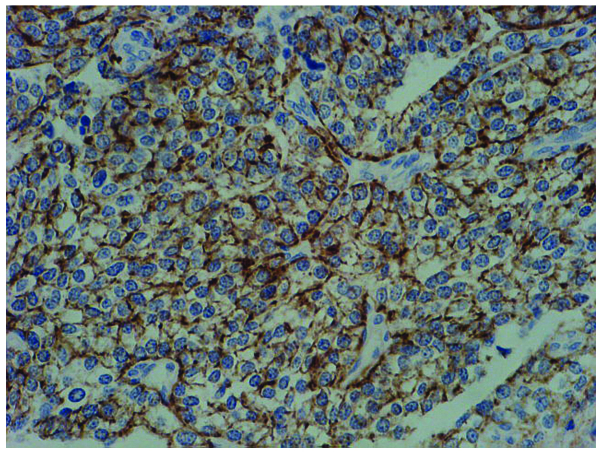
Immunohistochemical staining revealed high positive expression of cluster of differentiation 20 (hematoxylin and eosin; magnification, ×200).

A complete blood test revealed a white cell count of 6.21×10^9^ cells/l and the percentage of large white blood cells was 54.9%. The hepatic function (including serum lactate dehydrogenase levels) and renal function of the patient were unremarkable. The serum levels of carcinoembryonic antigen, neuron-specific enolase and CYFRA21-1 were also unremarkable. The results from the blood gas analysis were as follows: pH, 7.41; PaO_2_, 81.1 mmHg and PaCO_2_, 33.5 mmHg (under the condition of no oxygen inhalation). Chest CT scans revealed a mass in the right middle lobe of the lung with ground-glass opacities at the lesion margins, as well as air bronchograms in the areas of consolidation (Fig. 1). Bronchoscopy was performed and revealed an endobronchial lesion and partial stenosis in the distal end of the middle segment bronchus (Fig. 2). TBNA of the right hilar lymph node, as well as endobronchial biopsy, were performed (Fig. 3). The initial pathology of the specimens via endobronchial biopsy revealed infiltrative atypical lymphoid tissue that was highly suspicious for lymphoma; however, greater amounts of tissue were required for a definitive diagnosis. The following histopathological examination of pulmonary specimens collected via TBNA revealed an aggressive large cell lymphoma, with scattered large, oddly shaped nuclei resembling Reed-Sternberg cells (Fig. 4). Immunohistochemical staining was performed and revealed high expression levels of cluster of differentiation 20 (Fig. 5). Therefore, the patient was diagnosed with primary pulmonary DLBCL.

Following the diagnosis, a bone marrow biopsy was performed, which revealed normocellular marrow, and no granulomas or tumor (in particular no evidence of DLBCL) were detected. Furthermore, no evidence of a B-cell lymphoproliferative disorder was confirmed by flow cytometric analysis of the bone marrow aspirate. A complete enhanced CT scan of the abdomen and magnetic resonance imaging scan of the head showed no abnormalities. The patient was transferred to the department of hematology for CHOP chemotherapy treatment [cyclophosphamide 750 mg/m^2^ intravenously (i.v.) day 1, doxorubicin 50 mg/m^2^ i.v. day 1, vincristine 2 mg i.v. day 1 and prednisolone 100 mg orally days 1–5, which were planned to be repeated every 21 days for six cycles) following the diagnosis. Chest CT scans were performed following the administration of four cycles of CHOP chemotherapy, which showed no signs of disease. In addition, a marked improvement of the patient’s respiratory symptoms was observed. Complete remission was confirmed at the 8-month follow-up.

## Discussion

PPL is defined as clonal lymphoid proliferation affecting one or both lungs (parenchyma and/or bronchi) (6). PPL is extremely rare and accounts 3–4% of all Hodgkin’s lymphoma (HL) and <1% of all NHL (7,8 ). DLBCL is the most common PPL following MALT-type lymphoma, which occurs only in 10% cases of primary pulmonary NHLs (1,9). The pathogenesis of primary pulmonary DLBCL has yet to be clearly elucidated; however, a review of the literature indicates that it may be associated with long-term treatment using methotrexate (10) or immunosuppression, as observed in solid organ (heart/lung) allograft recipients on OKT3 or cyclosporin A, or in HIV infection (6,11).

To the best of our knowledge, patients with primary pulmonary DLBCL have no overt symptoms during the initial stages; however, as the disease progresses, they are likely to present with non-specific symptoms, including dyspnea, cough, chest pain, sputum and other obstructive and infectious symptoms, as well as fever and weight loss (6). As a consequence, the diagnosis of primary pulmonary DLBCL, in particular in a primary care setting, is challenging and often leads to misdiagnosis and delayed treatment, increasing medical costs and clinical risk (4).

With increasing awareness and improved diagnosis of primary pulmonary DLBCL, the reported incidence of the disease has increased significantly (1). Chest CT findings are various in primary pulmonary DLBCL and include solitary or multiple pulmonary nodules, masses, consolidation, hilar/mediastinal adenopathy, pleural effusion and, rarely, direct chest wall invasion (12,13). In particular, primary pulmonary DLBCL should be suspected in patients when masses or mass-like areas of consolidation and pulmonary nodules associated with findings of air bronchograms and a halo of ground-glass shadowing at lesion margins are detected.

Although the diagnostic procedure of this disease has not been well defined, open thoracotomy or chest CT-guided percutaneous biopsy are the preferred methods used in previous studies (14,15). Although the positive value of open thoracotomy biopsy is high, it is associated with high medical cost and clinical risk. It is not possible to perform this procedure in certain patients due to bad performance status. By contrast, the diagnostic value of chest CT-guided percutaneous biopsy is has a low medical cost and less serious complications. However, the procedure is limited for the diagnosis of masses that are in or adjacent to the hilum or mediastinum.

Although the use of bronchial endoscopy has been reported in previous studies, definitive diagnosis via direct bronchoscopic biopsy is rare, being achieved in only ~10% of patients reported in the previous studies (3–5). The diagnostic value of bronchial, and in particular transbronchial, biopsy is higher when it targets visible endobronchial lesions or radiographic abnormalities (16). Bronchoalveolar lavage (BAL) has also been reported; however, its value for the positive diagnosis of PPL has been inadequately assessed and requires further validation in larger prospective studies (6).

Since the introduction of TBNA in flexible bronchoscopy in 1983, conventional TBNA has been technically well established and its role has been expanded in the diagnosis and staging of lung cancer (17). To the best of our knowledge, the diagnostic value of TBNA in PPL has not been mentioned in previous studies. In the present case report, bronchoscopy was performed and an endobronchial lesion and partial stenosis in the distal end of the middle segment bronchus was observed. The initial pathology of the specimens via endobronchial biopsy revealed infiltrative atypical lymphoid tissue that was highly suspicious for lymphoma; however, a larger amount of tissue was required for a definitive diagnosis. Primary pulmonary DLBCL was confirmed following histopathological and immunohistochemical staining examination of a pulmonary specimen collected via TBNA. Therefore, TBNA may be an effective method for increasing the positive diagnosis rate of primary pulmonary DLBCL.

Methods for the treatment of primary pulmonary DLBCL, including simple monitoring, surgery, chemotherapy and chemotherapy followed by radiotherapy, are controversial and there is no uniform treatment strategy (6). However, treatment of primary pulmonary DLBCL is recommended to be based on biological features, including stage, histology and performance status (1). Seeram *et al* (4) reported that the majority of primarily pulmonary DLBCL is a low-grade malignancy and complete resection may be curative. However, the patient was not considered a surgical candidate due to an endobronchial lesion in the middle segment bronchus, and the patient preferred a conservative approach with chemotherapy alone. Following the final diagnosis, standard treatment with CHOP resulted in significant clinical and radiological response and the patient remained in remission 8 months later. This indicates that anthracycline-based chemotherapy may be an optimal therapeutic strategy for patients with primary pulmonary DLBCL (18,19).

In conclusion, primary pulmonary DLBCL is extremely rare with nonspecific signs and symptoms. TBNA, associated with endobronchial biopsy, may be an effective method for the diagnosis of the disease. Furthermore, CHOP chemotherapy may be an effective treatment for alleviating symptoms of the disease.
